# Febrile Seizures and Febrile Seizure Syndromes: An Updated Overview of Old and Current Knowledge

**DOI:** 10.1155/2015/849341

**Published:** 2015-11-30

**Authors:** Abdulhafeez M. Khair, Dalal Elmagrabi

**Affiliations:** Department of Pediatrics, Section of Pediatric Neurology, Hamad Medical Corporation, P.O. Box 3050, Doha, Qatar

## Abstract

Febrile seizures are the most common paroxysmal episode during childhood, affecting up to one in 10 children. They are a major cause of emergency facility visits and a source of family distress and anxiety. Their etiology and pathophysiological pathways are being understood better over time; however, there is still more to learn. Genetic predisposition is thought to be a major contributor. Febrile seizures have been historically classified as benign; however, many emerging febrile seizure syndromes behave differently. The way in which human knowledge has evolved over the years in regard to febrile seizures has not been dealt with in depth in the current literature, up to our current knowledge. This review serves as a documentary of how scientists have explored febrile seizures, elaborating on the journey of knowledge as far as etiology, clinical features, approach, and treatment strategies are concerned. Although this review cannot cover all clinical aspects related to febrile seizures at the textbook level, we believe it can function as a quick summary of the past and current sources of knowledge for all varieties of febrile seizure types and syndromes.

## 1. Introduction

Febrile seizures (FS) are among the most common reasons that patients present with to pediatric emergencies. These seizures are classically associated with high fever in children during their early lives [1]. Scientists used to think of FS as a benign condition, warranting nothing apart from reassurance. Over time, we have learned that the benign nature of FS should be carefully rethought, as there are a number of atypical presentations with variable outcomes [2].

## 2. Historical Background

FS has been recognized as a separate disease entity from other types of seizures since the early mid-nineteenth century. This was emphasized more after the invention of the thermometer in the late 19th century. Lennox was the first clinician to study the background and risk factors for FS and the risk of progression to epilepsy [3]. A few years later, the first community-based study was published, reviewing all convulsive disorders in young children and concluding that FS are probably benign and common and have good outcomes, but with a rare yet strong link to future epilepsy [4]. Pediatricians then started to recognize prolonged and recurrent FS as medical emergencies requiring more medical attention and urgent interventions; otherwise, future neurodevelopmental outcomes might be jeopardized [5]. The American Academy of Pediatrics' (AAP) committee of quality improvement published the first evidence-based practice parameters for FS [6]. The International League Against Epilepsy (ILAE) then developed a clearer consensus regarding the recognition and treatment of children with FS [7].

## 3. Epidemiology

Simple FS have an age range classically described as 6 to 60 months. The peak incidence is usually in the second year of life. FS are prevalent in up to 5% of children, with the overall incidence estimated to be 460/100,000 in the age group of 0–4 years [8]. Most FS are simple; however, up to 30% might have some complex features [9]. The risk of recurrence of FS is related to various factors, including younger age group, prolonged seizure duration, degree of fever, and positive personal and family history of FS [10]. In fact, a positive family history of FS in first-degree relatives is observed in up to 40% of patients [10]. Gender distribution has been studied in the literature. One previous study found a mild male predominance [11], but this has not been supported by other literature reviews. Seasonal variation with regard to seizure incidence has not yet been fully understood. Studies have shown that FS tend to occur more in the winter months and are more common in the evening [12]. The underlying pathophysiological explanations for these observations remain obscure.

## 4. Definitions

There are three chronological definitions currently used to characterize FS. The first definition was published in 1980 by the National Institutes of Health (NIH). It defined FS as an abnormal, sudden, excessive electrical discharge of neurons (gray matter) that propagates down the neuronal processes (white matter) to affect an end organ in a clinically measurable fashion, occurring in infancy or childhood, usually between 3 months and 5 years of age, and is associated with fever but lacks evidence of intracranial infection or defined cause [13]. The second definition was published by ILAE in 1993 and had the same concept, but it expanded the inclusion age group to young infants apart from neonates and excluded children with symptomatic febrile convulsions [14]. More recently, the American Academy of Pediatrics (AAP) has announced a standard definition of febrile seizures as a seizure occurring in febrile children between the ages of 6 and 60 months who do not have an intracranial infection, metabolic disturbance, or history of afebrile seizures [15].

## 5. Etiology and Pathophysiology

Signal pathway studies have only delivered theories in regard to why and how certain children develop FS. In the past, the most prevalent theory attributed a direct effect of hyperthermia on compensatory hyperventilation. This was assumed to cause mild brain alkalosis, resulting in increased neuronal excitability and the subsequent development of clinical seizures [16]. This theory, however, has not explained why some children are more prone to develop such phenomena than others. Currently we know that there is a large role of genetic susceptibility based on a large group of gene variants. This genetic makeup has likely resulted in neurodevelopmental vulnerability, with alterations in sodium channel expression, hypothalamic dysregulation, and both cortical and hippocampal excitability [17]. Environmental triggers, including nonfever causes, are then probably involved through neurotropicity and metabolic dysregulatory pathways [17].

## 6. Risk of First FS

Based on the previously mentioned causative theories, several risk factors for developing the first FS have been suggested. The degree of fever height is probably more relevant than the degree of rise of temperature itself, contrary to previous thought [18]. A history of FS in a first-degree or higher relative seems to be the factor with the strongest prediction power [19]. Other risk factors implicated in FS include preexisting developmental delays, day care attendance, stay in the neonatal nursery for more than 28 days, and various viral infections [19]. Associations with childhood vaccinations and some mineral deficiencies, such as zinc and iron, remain unclear at the moment.

## 7. Historical Milestones

### 7.1. Simple FS

The classical scenario is a short seizure in the setting of acute febrile illness other than central nervous system infection. It affects children between 6 months and 5 years of age. The seizure is described as generalized, lasting less than 15 minutes. The seizure semiology is either generalized clonic or generalized tonic-clonic. Seizures do not recur within the same febrile illness. The child is otherwise neurologically healthy, with no concerning focal neurological deficits. Motor and social development is usually normal [20]. History and physical examination are vital to determine the cause of the fever. There are no routine laboratory tests needed, but a check of electrolytes and blood sugar levels might be warranted, especially with a gastroenteritis illness. CSF studies should be considered for the youngest age group (less than 18 months old), as definitive signs of CNS infections are often difficult to judge [21]. Neuroimaging studies are reserved for patients with a history of trauma or unusual residual neurological manifestations [22].

Treatment trials for FS belong to an old history in the medical literature. In the late 1970s, scientists thought of treating children with regular antiseizure medications after their first FS. The initial randomized controlled trial (RCT) by Camfield and his group in Canada described the use of phenobarbital in a population of 102 patients [23]. The patients in this study were assigned to treatment and placebo groups. The study concluded that daily use of phenobarbital reduced the rate of subsequent FS from 25 to 5 per 100 subjects per year. Nevertheless, 50% of patients had been noncompliant, and nearly 40% had experienced significant side effects [23]. Another blinded RCT by Ngwane and Bower from Oxford a few months later assessed the usefulness of sodium valproate in the prevention of simple FS [24]. In this trial, patients were assigned to phenobarbital, valproate, and placebo groups. It was found that only 4% of children in the valproate group had further seizures, as opposed to 35% in the control group. The study concluded that valproate was very effective in treating FS and that no life-threatening side effects were encountered [24].

A few years later, researchers started to entertain the concept of giving an intermittent shorter course of antiseizure medications to children at risk during the febrile illness in order to prevent further FS episodes. The largest study of this group was a double-blinded RCT by Rosman and his group, involving a trial of intermittent diazepam therapy during the febrile illness for at-risk children [25]. This was a large study, with more than 400 patients enrolled and assigned to treatment and placebo groups. The results showed a significant reduction in seizure recurrence with relative risk of 0.18 [25]. A major criticism for this study, however, was that the number of patients to treat to prevent one FS was 14. Many clinicians consider this unacceptable [25].

After the publication of the intermittent diazepam study, a new line of thinking was more benign, using regular antipyretics instead of antiseizure medications for FS prevention in risky patient groups. Schnaiderman and his group from Israel conducted a large RCT of more than 100 previously healthy children [26]. They then divided the patients into three groups: regular acetaminophen, on-demand acetaminophen, and placebo. Neither the risk of seizure occurrence nor recurrence differed between the three groups. The study concluded that use of regular or intermittent antipyretics did not seem to be helpful, and the classical fever management advice to parents had to be reviewed and updated [26].

The concept of rescue seizure medication arrived in the research world approximately 10 years ago. The largest study was by Scott from the UK, who studied children with FS between the ages of 1 to 4 years, excluding young infants [27]. He compared rectal diazepam versus buccal midazolam as an abortive seizure medication in a cohort of all patients who presented to emergency services with prolonged seizures of more than 5 minutes' duration. In his study, midazolam aborted 54% of all seizure types, whereas diazepam aborted 27%, with good statistical significance [27]. The study concluded that buccal midazolam is superior and can be considered the drug of choice for aborting prolonged seizures, including FS [27]. Last year, the Camfield family performed a meta-analysis to assess candidacy for choosing the ideal candidate for rescue antiseizure medications [28]. According to her review, children with a history of prolonged FS lasting more than 10 minutes, patients who belong to anxious parents, and patients with logistic difficulties accessing the health care facility are the ideal candidates for rescue FS medication [28].

### 7.2. Complex Febrile Seizures

This group represents FS with any atypical features. Those features include age group outside the usual range, focal onset seizures, prolonged seizures lasting more than 15 minutes, recurrent seizures within the same day, and patients with an unexpected prolonged recovery period.

The age group distribution initially received considerable attention from researchers. In the late 1980s, Pavone and his group from Italy followed 222 patients with their first FS happening after the age of 6. Of these patients, 42% had subsequent febrile and afebrile seizures [29]. He estimated the risk of recurrence for FS in this group at 36%, whereas the risk of future epilepsy was 15%. This is quite high in comparison to the risk in the general population [29]. A few years later, Berg and Shinnar followed a larger cohort of 686 patients with complex FS [9]. They found a unique correlation between low temperature upon presentation, longer seizures, and prolonged recovery. Interestingly, their study did not make any conclusions in regard to an increased risk of future epilepsy [9].

## 8. Risk of Epilepsy

Determining whether FS are able to convert to frank epilepsy has always been in the mind of pediatricians and researchers. The first national population-based study came from the UK and found no difference in the risk of subsequent epilepsy after simple FS compared to the general population [30]. In the late 1990s, we learned that complex features of FS and a strong family history of epilepsy might carry a significantly higher risk of future epilepsy [31]. Later, Tsai and Hung found that the presence of more than one risk factor, especially a preexisting neurodevelopmental disease, a positive family history of epilepsy, or two or more complex features, may increase the epilepsy incidence fourfold [32]. Although the risk factors for recurrence of FS are quite different from the risk factors of subsequent epilepsy, one interesting study found that the risk of epilepsy may be slightly elevated with simple FS if they were very recurrent [33].

## 9. Mesial Temporal Sclerosis (MTS)

An anatomical explanation for the unclear link between FS and epilepsy has been explored as well. The anatomical finding with the highest level of interest was MTS. Although there seems to be a strong correlation between MTS and childhood FS [34], it is still uncertain which came first. It is not yet definitively known if FS causes MTS, which can trigger further epileptic episodes, or whether MTS leads to both FS and epilepsy, especially temporal lobe epilepsy (TLE). The most likely explanation is that both complex FS and MTS have a shared genetic background, which should be examined in further studies [35].

The prediction of epilepsy evolution after FS via other neurophysiological or radiological studies has also been reviewed. The largest study to date is the Korean EEG study, which has followed up more than 1000 children with FS over a 5-year recruitment period [36]. In that study, 12% of the 183 patients with complex FS developed overt epilepsy. Focal epileptiform discharges have been elicited more frequently and significantly in the epilepsy group, more than five times higher than in other groups [36]. A major limitation of this large study, however, was that it was retrospective and single centered. A larger and better-designed study has recently been published by Berzosa Lópe and his group from Spain [37]. They have studied a cohort of more than 3000 patients with complex FS over 9 years, excluding those with any previous neurological illness. Interestingly, the neuroimaging studies were entirely normal in all patient populations [37]. Moreover, the results of EEG recordings could not be linked to risk of future epilepsy in that cohort [37]. The study has concluded that the incidence of complications in complex febrile seizure in this series of patients did not justify either the systematic admission or the systematic study with complementary tests when the neurological examination was normal [37].

## 10. Generalized Epilepsy with Febrile Seizures (GEFS+)

This ill-defined category is basically a heterogeneous familial syndrome with patients displaying FS, often after the age of 6 years, in addition to a variety of afebrile seizure types. It is assumed to be inherited through an autosomal dominant pathway [38], though its genetic background is more complex than can be explained by simple Mendelian inheritance. Seizures vary in their frequency, semiology, and response to treatment. Neuroimaging is usually normal in most if not all patients. Occasionally, some patients are intellectually challenged, which leads to questioning whether the whole disorder is to be considered one of the epileptic encephalopathies [38].

The neurogenetics of GEFS+ have always been an exciting and growing field of research. Currently, most experts in molecular genetics classify GEFS+ into three groups based on the underlying genetic makeup. GEFS+ type 1 is usually linked to SCN1B gene mutation, GEFS+ type 2 to SCN1A, and GEFS+ type 3 to GABRG 2 gene mutation [39]. The latter is the only gene that encodes sodium channels. The most mysterious among those genes is the SCN1A beta subunit, which is located in 2q24.3 and linked to GEFS+ type 2. This sodium channel-encoding gene has been linked to many neurological syndromes of variable clinical spectra and severity. Examples of these syndromes include early infantile epileptic encephalopathy (Ohtahara syndrome), severe myoclonic epilepsy of infancy (Dravet syndrome), intractable childhood epilepsy with generalized tonic-clonic seizures (ICE-GTC), myoclonic astatic epilepsy (Doose syndrome), malignant migrating partial seizures of infancy, and familial hemiplegic migraine type 3 [40].

## 11. Febrile Infection Related Epilepsy Syndrome (FIRES)

This syndrome constitutes another emerging disease entity that is closely related to FS and epilepsy. In their multicenter study on 77 patients with FIRES, Kramer et al. estimated its prevalence to be 1 : 100000 children, but many think this number might be an underestimate [41]. Most patients are between 3 and 15 years old, and boys are affected more than girls. Although the disease is thought to be familial, no more than one case has been reported from the same family. The etiology is not exactly known; the proposed inflammatory and immunological factors have not been confirmed yet. There is currently no clear-cut evidence correlating FIRES with mitochondrial dysfunction [42].

Seizures tend to happen solely during febrile illnesses, but they are quite explosive, prolonged, and lifelong. Most seizures are focal at the beginning, but an evolution to generalized seizures is common. Many patients develop a number of neurological symptoms over time, including learning and motor difficulties, behavioral changes, nonspecific sensory symptoms, and memory deficits [41]. EEG studies often show generalized slow background with ictal frontal and temporal epileptiform activity. Brain imaging studies are initially normal but over time demonstrate progressive brain atrophy with or without temporal hyperintensities [42].

Antiepileptic medications are often ineffective. High doses of benzodiazepines might offer some control, but they have a number of side effects. Burst suppression might be needed. The role of immunotherapy is questionable, though Sakuma et al. have reported a success rate of 85% with steroids [43]. In their study, however, there was no response to intravenous immunoglobulins (IVIG), making the conclusive immunotherapeutic benefit rather difficult [43]. The syndrome is occasionally fatal, and the overall prognosis is poor. One possible promising treatment option is the ketogenic diet, which has been reported to have some success in a few case reports. The largest series of these is the series of seven patients by Nabbout et al. from France [44]. In their patients, the ketogenic diet has led to a 50% reduction in seizure frequency in the first week of treatment in all patients with FIRES [44]. This study, however, lacks long-term follow-up, which limits whether the findings can be added to the treatment recommendations.

## 12. Febrile Status Epilepticus (FSE)

This condition describes prolonged FS lasting more than 30 minutes in duration. Most of our current knowledge about this condition comes from the famous FEBSTAT study [45]. The peak age is between 12 and 24 months, and this condition is very unusual after 5 years [45]. Some studies have described an average incidence of 4 per 100000 per year [45]. There is an unexplained increased prevalence in children of Asian descent. Two-thirds of seizures are generalized, and one-third are of focal semiology. There is no clear reason why some children tend to have prolonged FS and others do not. Magnetic resonance imaging (MRI) studies show hippocampal swelling in half of patients from the third day of seizure [46]. Whether this is the start of the process evolving into TLE is still an ongoing scientific debate.

It has been observed that antipyretics do not seem to shorten seizure duration [47]. The major stay of treatment relies on routine seizure abortive measures and exploring the source of fever, with special attention on the possibility of CNS infection. There is no conclusive evidence that febrile status epilepticus in a previously healthy child can increase the risk of future epilepsy [47]. The latest review in this area described the demographics and outcomes and was published by Nishiyama et al. from Japan [48]. This recently published study of cohort of 253 children with FSE has identified poor prognostic factors as male gender, body temperature above 40 degrees C upon presentation, seizure duration of more than 3 hours, and the presence of nonconvulsive status epilepticus [48].

## 13. Afebrile Febrile Seizures (AFS)

This is the newest terminology that has arrived in the world of FS science. AFS appears to be a distinct disorder that is not linked to other FS syndromes. This disorder refers to children who have provoked seizures lacking objective evidence of fever at the seizure onset but have definitive symptoms and signs of minor infection. The presenting illness is usually a mild respiratory or gastrointestinal infection. An example of a gastroenteritis-related infectious agent is rotavirus, which has been linked in many studies to both febrile and afebrile childhood seizures [49]. A number of respiratory viruses have served as etiological agents for FS, influenza A being one example [50]. Seizures mostly occur in the first three days of life.

The risk of subsequent epilepsy in this group of children is much higher than that in the normal population and is thought to be approximately 7.5% [51]. This assumption, however, has been challenged by newer studies such as Lee and Kim study from Korea. Lee and Kim followed 120 children with provoked seizures over five years, allocating them into febrile and afebrile groups. Their study has concluded that the risk of epilepsy is low and not significantly different between the two groups, with or without the presence of fever [52]. This concept has been dissected further by Zhang et al. from China in their large prospective study [53]. This was the first study to compare provoked seizures induced by gastrointestinal versus respiratory illnesses and contrasting them with unprovoked epileptic seizures. The new information from this study is that gastrointestinal illness-related seizures have a low risk of recurrence and good outcomes. Respiratory illness-related seizures, in contrast, have a similar risk of future epilepsy as unprovoked seizures and a worse overall prognosis [53].

## 14. Vaccinations-Related FS

The relationship between childhood vaccines and febrile seizures has attracted attention of the media and medical fields. Although the correlation between the two is difficult to describe as incidental, researchers have discovered that FS after vaccinations are no different from FS of other causes [54]. The risk of hospitalization and illness course are not different between vaccination-related and other illness-related FS [55]. It is worth mentioning that postvaccination FS are quite rare and often occur within the first three days after administration of live attenuated vaccines. Concomitant multivaccination administration is believed to increase the risk of developing FS [56].

There is no current evidence of any increased risk of either subsequent seizures or neurodevelopmental affection after the initial seizure. It is thus very crucial to alert the families to the fact that none of the standard vaccinations is currently contraindicated in children with FS. Prescribing fever-lowering medication around the time of some potentially pyrexic vaccinations can be a reasonable practice in children at risk of FS [57]. Currently, there is not enough evidence to suggest the usage of other rescue medications as an FS preventive measure after vaccination for at-risk children [58].

## 15. Ongoing and Future Research

There is much work in progress to further understand FS. A large ongoing north London status epilepticus in childhood surveillance study (NLSTEPSS) is running, evaluating long-term morbidities and treatment options for FS. Initial short-term outcomes were released in 2006 [59]. Some basic science studies sponsored by the United States National Institutes of Health (NIH) are working to understand the mechanisms of possible epileptogenesis of FS, using animal models. Some of the initial results have been published recently [60]. The most exciting project in the area of FS is probably the FEBSTAT study. This study is examining the pathophysiology and the long-term clinical, electrical, and radiological consequences of FSE. Very rich data have been achieved, and some valuable results have been published recently. Examples of these data are future developmental outcomes after FSE [61] and possible correlations with seizure duration [62]. The possible contribution of certain viruses has been highlighted as well [63]. A more in-depth look at acute electrophysiological [64] and laboratory [65] changes after FSE has been noted. The genetic makeup of special FS syndromes is expected to lead the revolution in improving our understanding of FS. A more updated genetic dissection of FS syndromes has been established by a recent twin study [66]. Some light has been shed on the roles of synaptic transmission [67], potassium [68], and sodium channelopathy [69] in the pathogenesis of several FS syndromes. Genetic sequencing and variant analysis appear to be the sophisticated future of investigating FS genetic syndromes [70].

## 16. Conclusion

Although febrile seizures are commonly benign, most families consider them very frightening. This puts pressure on clinicians to accurately reassure families by being equipped with the recent up-to-date knowledge. It is important to realize some special febrile seizure syndromes, which can have some long-term neurological abnormalities. An extensive medical workup is often not needed, provided that possible serious CNS infection is considered. There is a great journey of knowledge of FS occurring in the area of advanced genetic and functional studies. This review serves as a summary of the available evidence in regard to FS categories and how knowledge has progressed over the years. More studies are still needed to help the medical community's understanding of the mechanisms, pathways, correlations, and clinical implications of FS and FS-related syndromes.
